# The Effect of Sleep Disorders on Sexual Function in Bipolar Disorder in the Remission Period

**DOI:** 10.1192/j.eurpsy.2022.311

**Published:** 2022-09-01

**Authors:** M. Ünlü Çilesiz, N. Tomruk, N. Çilesiz, C. Varlık, E. Yıldızhan

**Affiliations:** 1 Bakirkoy Prof. Dr. Mazhar Osman Mental Health and Neurological Diseases Training and Research Hospital, Psychiatry 14, İstanbul, Turkey; 2 Biruni University Faculty of Medicine, Urology, İstanbul, Turkey

**Keywords:** Bipolar Disorder, Sleep Disorder, Sexual Dysfunction

## Abstract

**Introduction:**

Bipolar Disorder(BD) is a common,severe and recurrent disease with significant effects on functionality. Residual symptoms such as sleep disturbance and sexual dysfunction are defined as predictors of poor functioning in remission.

**Objectives:**

The aim of this study was to investigate the correlation between sleep disorder and severity of sexual dysfunction in patients with BD in remission.

**Methods:**

The study was conducted with 100 female and 100 male BD patients in remission. The sociodemographic and clinical characteristics were recorded by interview with the patients and the patients were given the Young Mania Rating Scale(YMRS),Hamilton Depression Rating Scale(HAM-D),Pittsburg Sleep Quality Index(PSQI),Epworth Sleepiness Scale(ESS),Female Sexual Function Scale(FSFI) and International Index of Erectile Function(IIFF-15)for the assesment of symptom severity.

**Results:**

The frequency of “sleep disorder” was 45.5% and the frequency of ’’daytime sleepiness’’ was 5.5%.In women the mean FSFI score was 26.06±5.14 and sexual dysfunction frequency was 48%.In men,the mean IIEF score was 59.63±8.34 and erectile dysfunction frequency was 56%. There was a statistically significant negative correlation between total FSFI score with HAM-D(r=-0.592, p <0.001),ESS (r=-0.330, p=0.001)and PSQI(r=-0.557, p<0.001)and between total IIEF score with HAM-D(r=-0.509, p<0.001),ESS(r=-0.361, p<0.001)and PSQI(r=-0.511,p<0.001). Sexual function scores in both women and men with sleep problems were significantly lower than those without sleep problems(23.56±4.71vs.28.56±4.31and53.88±7.10vs.63.80±6.56 respectively). Multiple linear regression analysis also showed that total sleep quality scores were an effective factor on sexual function in women(OR:2.74,%95CI[0,799-0,127];p=0,007) and men(OR:2.45, %95CI[1.577-0.164];p=0,016) with BD.

**Conclusions:**

There was an increased incidence of sexual dysfunction in bipolar patients with sleep disorders.Treatment of sleep disorders is important for improving sexual function in bipolar patients for both genders.

**Disclosure:**

No significant relationships.

